# A Pro-Inflammatory Signature Constitutively Activated in Monogenic Autoinflammatory Diseases

**DOI:** 10.3390/ijms23031828

**Published:** 2022-02-05

**Authors:** Paola Galozzi, Ola Negm, Sara Bindoli, Patrick Tighe, Paolo Sfriso, Leonardo Punzi

**Affiliations:** 1 Rheumatology Unit, Department of Medicine DIMED, University of Padova, 35128 Padova, Italy; paola.galozzi@unipd.it (P.G.); sara.bindoli@phd.unipd.it (S.B.); paolo.sfriso@unipd.it (P.S.); 2 School of Life Sciences, The University of Nottingham, Nottingham NG7 2UH, UK; ola.negm@nottingham.uk (O.N.); paddy.tighe@nottingham.uk (P.T.); 3 Medical Microbiology and Immunology Department, Faculty of Medicine, Mansoura University, Mansoura 35516, Egypt; 4 Center for Gout and Bone & Joint Metabolic Diseases, SS Giovanni & Paolo Hospital, 30122 Venice, Italy

**Keywords:** monogenic autoinflammatory diseases, signature, inflammation, NF-κB pathway, MAPK pathways, PI3K–Akt pathways, Th17-related cytokines

## Abstract

Autoinflammatory diseases (AIDs) are disorders characterised by recurrent inflammatory episodes in charge of different organs with no apparent involvement of autoantibodies or antigen-specific T lymphocytes. Few common clinical features have been identified among all monogenic AIDs (mAIDs), while the search for a common molecular pattern is still ongoing. The aim of this study was to increase knowledge on the inflammatory pathways in the development of mAIDs in order to identify possible predictive or diagnostic biomarkers for each disease and to develop future preventive and therapeutic strategies. Using protein array-based systems, we evaluated two signalling pathways known to be involved in inflammation and a wide range of inflammatory mediators (pro-inflammatory cytokines and chemokines) in a cohort of 23 patients affected by different mAIDs, as FMF, TRAPS, MKD, Blau syndrome (BS), and NLRP12D. Overall, we observed upregulation of multiple signalling pathway intermediates at protein levels in mAIDs patients’ PBMCs, compared with healthy controls, with significant differences also between patients. FMF, TRAPS, and BS presented also peculiar activations of inflammatory pathways that can distinguish them. MAPK pathway activation, however, seems to be a common feature. The serum level of cytokines and chemokines produced clear differences between patients with distinct diseases, which can help distinguish each autoinflammatory disease. The FMF cytokine production profile appears broader than that of TRAPS, which, in turn, has higher cytokine levels than BS. Our findings suggest an ongoing subclinical inflammation related to the abnormal and constitutive signalling pathways and define an elevated inflammatory cytokine signature. Moreover, the upregulation of Th17-related cytokines emphasises the important role for Th17 and/or Th17-like cells also in monogenic AIDs.

## 1. Introduction

Autoinflammatory diseases (AIDs) are a heterogeneous group of disorders characterised by immune antigen-independent dysregulation conditions typically presented in childhood with fever and sterile inflammation [1]. Some common clinical features have been identified between all inherited AIDs, such as recurrent inflammatory episodes, the presence of fever, and frequent involvement of skin, joints, gastrointestinal tract, nervous system, and other tissues. However, each syndrome may have more or less severe inflammatory manifestations [2]. The rarity of these disorders often leads to their misunderstanding, with delayed diagnosis and treatments.

**Figure 1:**
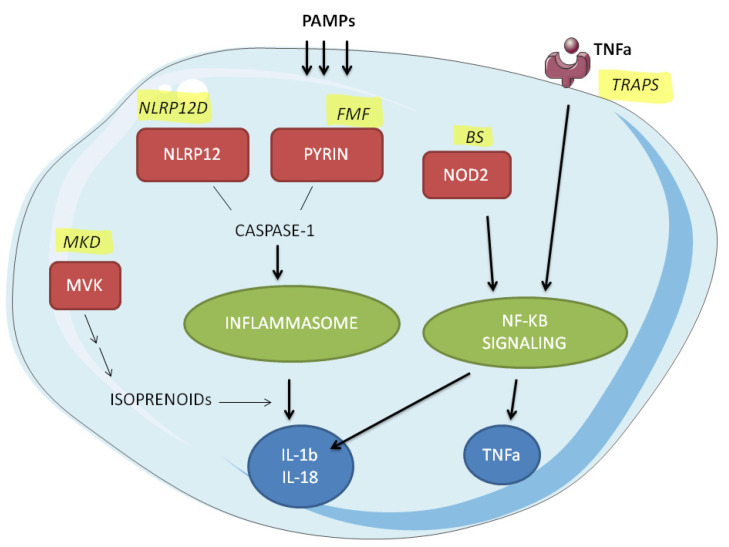
A simplified scheme of the pathogenesis of the monogenic autoinflammatory syndromes considered in this study.

The genetic cause of some familial autoinflammatory syndromes was identified more than 20 years ago [3,4]. Since then, the number of new monogenic autoinflammatory diseases (mAIDs) has been increasing every year. Owing to advances in genome research and technology, understanding of the pathogenesis of mAIDs has also grown rapidly over the past decade [5]. The genes responsible for mAIDs have been found to encode key sensors and transducers of inflammatory signalling pathways [6]. Some of the genes involved, such as *TNFRSF1A*, were previously well known, while the discovery of mutations in *NLRP3*, *NLRP12*, *NOD2* highlighted their importance as inflammatory signalling pathways (Figure 1). Indeed, mAIDs pathogenesis can result from dysregulated cytokine production mediated by the inflammasome, defective clearance, and regulatory pathways such as NF-κB and interferon (IFN)-related signalling, impaired protein folding, and intracellular stress [7]. The first step of the inflammatory response is the recognition of pathogens (PAMPs) by appropriate sensors (including NLR domains and pyrin), with the formation of a multimeric protein complex, called the inflammasome. The inflammasome receptors interact with the adapter protein ASC, leading to the activation of caspase 1, which converts pro-IL-1 and pro-IL-18 to their bioactive forms. This is one of the key signalling pathways that control the innate immune response. Furthermore, PAMPs are sensed by Toll-like receptors (TLRs), mainly TLR-4 or NOD2 receptors, and activate the NF-kB pathway, enhancing NLRP3 transcription and promoting a positive feedback effect [5]. Alterations at different levels of these complex mechanisms are associated with the development of different mAIDs. Moreover, this molecular heterogeneity could be directly related to the type of pathogenic variant. Null alleles or mutations in functionally critical residues can cause severe manifestations, while polymorphisms or hypomorphic mutations lead to a mild or late-onset form of mAIDs [6]. With the increasing knowledge about genotype–phenotype correlations, genetic heterogeneity and digenic inheritance could be included in overlapping phenotypes, which have been described for AIDs patients [8].

Although common clinical features of mAIDs have already been identified, a general molecular pattern that can characterise the spectrum of the mAIDs remains poorly understood. The mechanism of action of each gene associated with classical mAIDs has been extensively described in a recent review [9].

**Figure 2:**
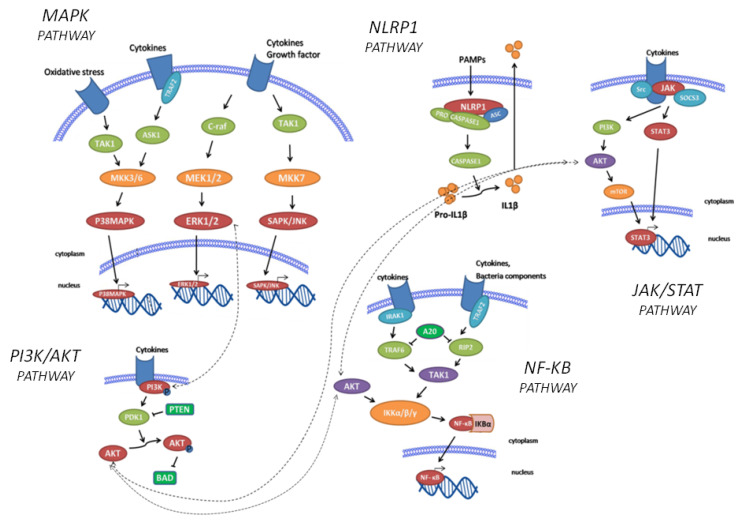
Schematic representation of the 5 signalling pathways considered in this study: NF-κB, PI3K–AKT, MAPK, JAK–STAT, and inflammasome (NALP1) pathways. It does not show every target examined in this paper. Dashed lines represent interactions between pathways.

In this study, we extended the analysis to five inflammatory signalling pathways (Figure 2), evaluating both signalling intermediates and inflammatory mediators with multiplex analytical arrays. Thus, increasing the knowledge on the possible molecular mechanisms involved in phenotypic variability in mAIDs will pinpoint potential predictive or diagnostic biomarkers and novel targets for ameliorated therapeutic interventions.

## 2. Results

To evaluate the cytokine profile and to identify the differential activation of the inflammatory pathways in familial Mediterranean fever (FMF), TNF-receptor associated periodic syndrome (TRAPS), mevalonate kinase deficiency (MKD), Blau syndrome (BS), and NLRP12 deficiency (NLRP12D), we used a protein array-based system with high sensitivity. We focused on a wide range of compounds that are known to play an important role in inflammation.

For reliable results, the optimisation of the multiplex assay conditions was required [10], in terms of the printing surface, spot array design, buffers, fluorescent signal amplification, and detection method.

### 2.1. Inflammatory Signalling Pathways

**Figure 3:**
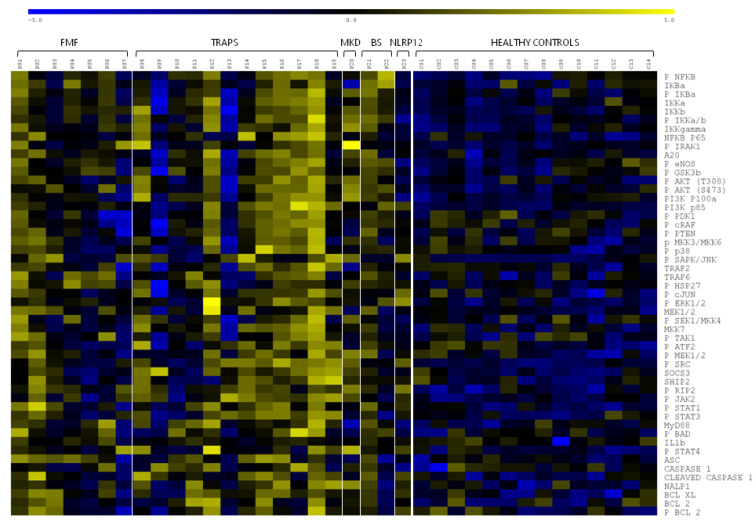
Heatmap of the pathway intermediates determined by RPPA in patients PBMCs. The multiple different proteins are outlined on the horizontal axis, and the lysates phenotype is on the vertical axis. Yellow and blue colours indicate high and low protein expression, respectively.

**Figure 4:**
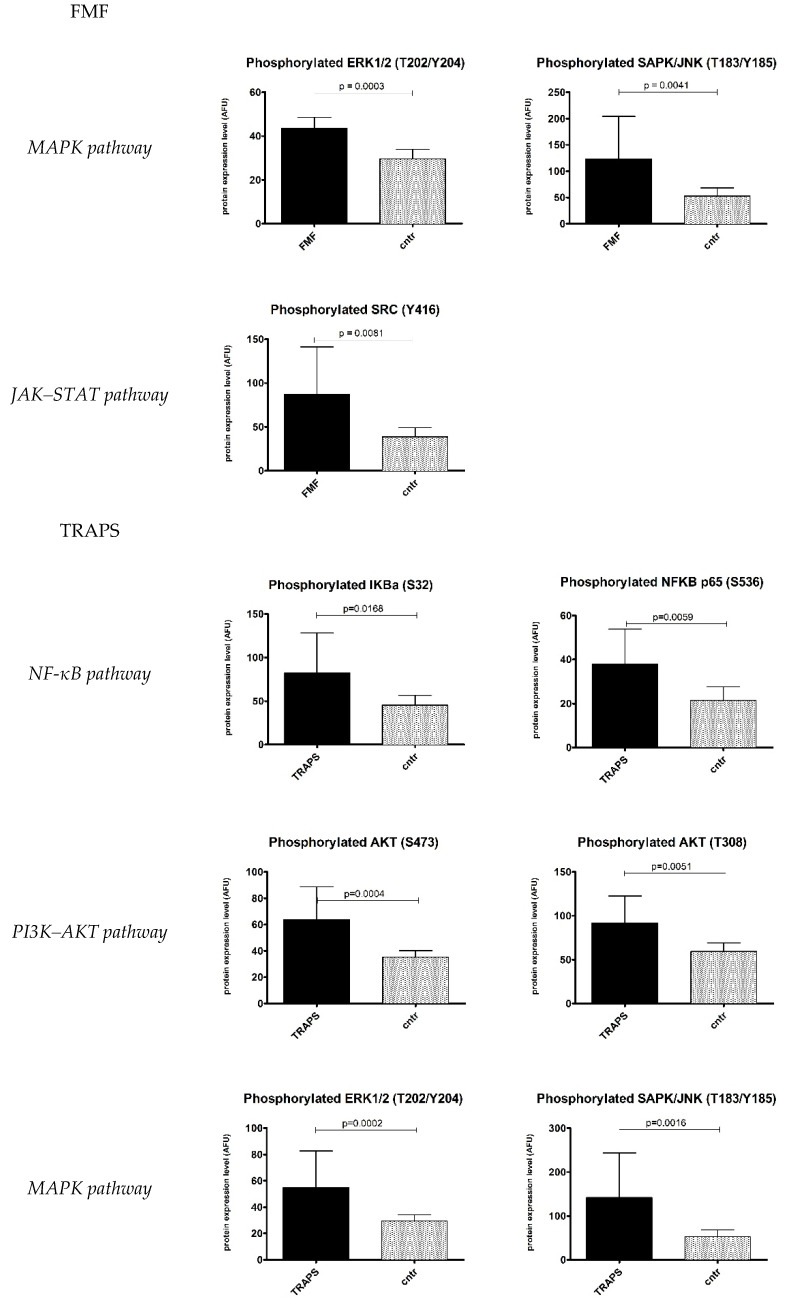

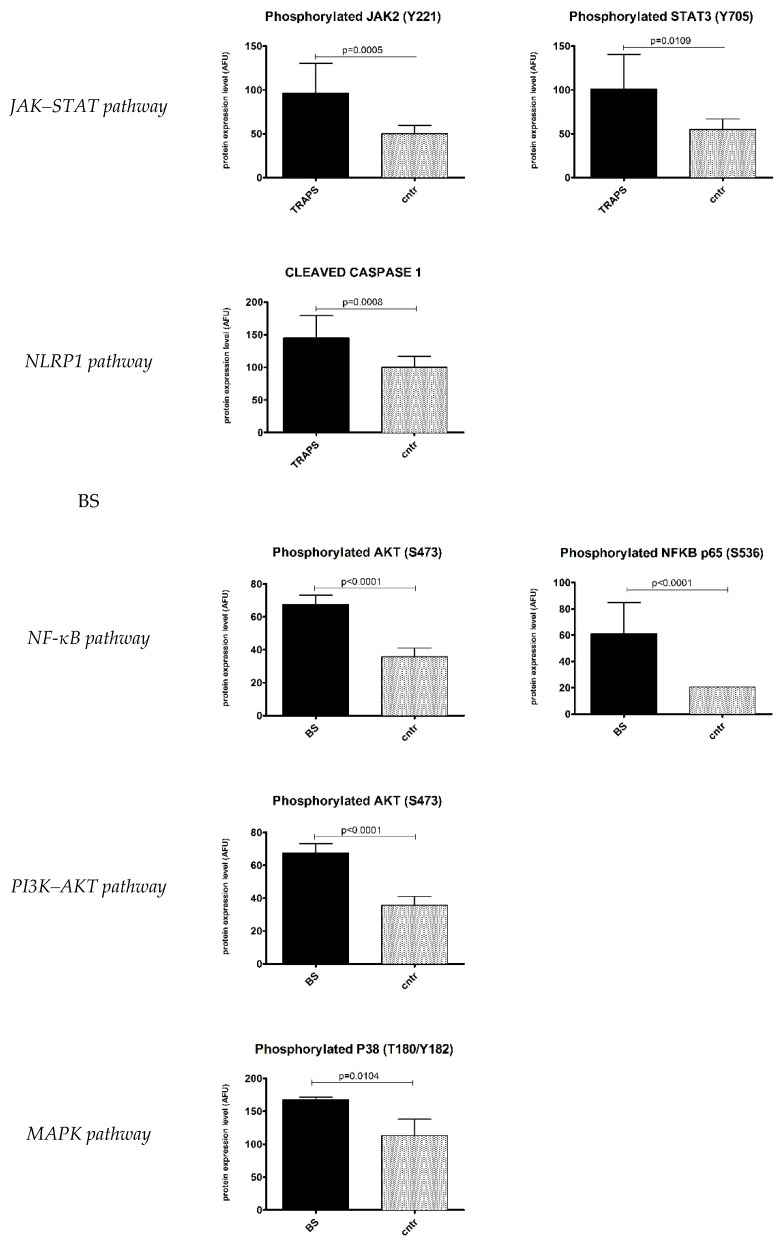
Significantly upregulated protein expression levels of the key intermediates in their active forms for each pathway (NF-κB, PI3K–AKT, MAPK, JAK–STAT3, and NLRP1 pathways) in FMF, TRAPS, and Blau syndrome (BS) patients (black bar), compared with healthy controls (dotted bar). The fluorescent signals are reported as arbitrary fluorescence units (AFU), with β-actin normalisation. Data are shown as means ± SD of three independent experiments. Cntr = healthy controls.

Peripheral blood mononuclear cells (PBMCs) from mAIDs patients and sex- and age-matched healthy controls were purified and proceeded, as described in the Materials and Methods section. Protein expression levels of key components of NF-κB, PI3K–AKT, MAPK, JAK–STAT, and NLRP1 inflammasome signalling pathways (Figure 2) were measured by reverse-phase protein array (RPPA). The expression levels normalised to β actin for each disease are shown in Figure 3 as a heatmap of the log 2 relative expression levels detected for each sample. The significant expression levels are also shown in plots (Figure 4) for selected key mediators as mean ± SD of three different biological replicates. Bar plots refer only to those disease groups with more than one patient (FMF, TRAPS, and BS). The protein expression levels of all upstream and downstream significant pathways components are graphed in Supplementary Figures S1–S3.

Overall, the RPPA results revealed that the majority of the analysed proteins and their phosphorylated forms were constitutively upregulated in TRAPS patients, compared with healthy controls. FMF and BS, instead, showed different and specific pathway activations, suggesting a kind of molecular signature. An interesting result to note is that the MAPK pathway was activated in all three diseases.

Moreover, the heatmap can suggest that the patterns of the signalling compounds may relate also to individual patients, as observed for TRAPS. In our cohort, patients 9, 10, 11, and 13 presented relatively low expression levels across virtually almost all pathways.

Despite the absence of supporting statistical analysis, Figure 3 can also help us derive observations for MKD and NLRP12D. It appears to be an activation of all five pathways in the MKD patient, while the expression levels of the NLRP12D patient are comparable to those of healthy controls.

### 2.2. Upregulated Pro-Inflammatory Cytokines

**Figure 5:**
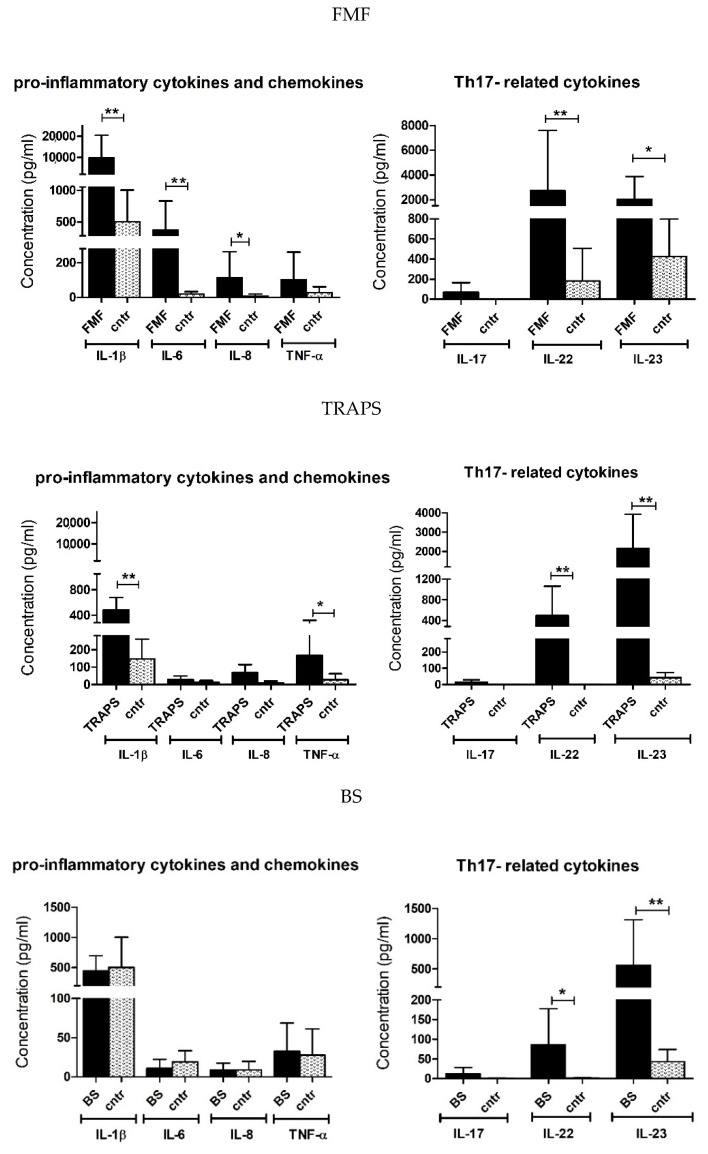
Cytokine profile from mAIDs patients and controls serum, obtained with antibody microarray technique. Data are means of triplicate experiments results and the error bars indicate standard deviation. * *p* value < 0.05; ** *p* value < 0.005; cntr = healthy controls.

The levels of cytokines and other markers of inflammation (IL-1β, IL-6, IL-8, TNF-α, IL-17, IL-22, and IL-23) have been assessed through antibody microarray in serum samples. As for RPPA, plots are referring only to those disease groups with more than one patient (FMF, TRAPS, and BS) (Figure 5).

Overall, most cytokine levels significantly raised in FMF and TRAPS, compared with controls. In FMF patients, significantly increased concentration levels were reported for IL-1β, IL-6, IL-8, IL-22, and IL-23, presenting, respectively 19-fold, 19-fold, 12-fold, 15-fold, and 5-fold higher values than the controls. In TRAPS patients, IL-1β, TNF-α, IL-22, and IL-23 levels significantly increased more than controls. In particular, extremely high concentrations were found for IL-22 and IL-23, which increased by more than 200 and 50 times, respectively, compared with the controls. In BS patients, only IL-22 and IL-23 were higher expressed than the controls (80-fold and 13-fold, respectively). In all disease groups, therefore, it is particularly striking the upregulation of the Th17-derived cytokines (IL-22 and IL-23), suggesting an important role for Th17 or Th17-like cells in mAIDs.

The resulting cytokine pattern may be of help to distinguish or stratify the mAIDs in terms of the number of cytokines raised. Indeed, the FMF cytokine profile seems broader than that of TRAPS, which, in turn, presented higher cytokines levels than BS.

## 3. Discussion

Several genome-wide association studies have begun to elucidate the molecular basis of autoinflammatory diseases. Most of these studies underline the prominence of the IL1β-activating inflammasome and its regulation in a large number of AIDs. More recently, additional mechanisms linking innate immune-mediated inflammation with a variety of cellular processes, including protein misfolding, oxidative stress, and mitochondrial dysfunction, have been recognised to play roles in the pathogenesis of certain monogenic autoinflammatory conditions [5,11,12,13]. These processes can later activate other inflammatory pathways with host defence function, such as MAPK, JNK, and NF-κB pathways. Despite these major advances, not all patients show the expected clinical characteristics or respond favourably to drugs, leading to high morbidity in these diseases.

Based on this premise, our study aimed to improve our understanding of how multiple inflammatory signalling pathways and cytokine profiles affect different mAIDs. Identifying such patterns is a sizeable problem, but using an integrative approach to analyse a great number of possible biomarkers represented an enormous advance. To enable this, microarray-based systems have been the high-throughput assays to measure cytokines and report signalling intermediates’ levels. RPPA was able to provide a descriptive picture of the ongoing state of signalling networks and gave information about the post-translational modifications such as phosphorylation that cannot be provided using gene study [14]. The antibody microarray, on the other hand, was a highly sensitive and specific test that allowed highly sensitive detection of cytokine levels, even in case they were not very abundant.

Overall, we observed a generalised and constitutive increase in protein levels for several proinflammatory signalling intermediates (phosphorylated or not) in mAIDs patients’ PBMCs. More specifically, the three diseases with more substantial cases in our cohort (FMF, TRAPS, and BS) then present characteristics that can distinguish them. Although the majority of mAIDs patients were under therapy and generally with controlled disease symptoms, altered cytokine patterns and activated pathways support the idea of subclinical and constitutive inflammation.

In FMF patients, only MAPK (ERK and SAPK) and JAK–STAT pathways were activated, even though the literature data are discordant. Indeed, it has been reported that pyrin can activate the NF-κB pathway and can be regulated in a MAPKp38-dependent mechanism [15,16]. A possible explanation lies in our cohort, which consists of patients with a mild form of FMF and an adult-onset of the disease. Interestingly, the activation of the JAK–STAT pathway could shed light on the use of JAK inhibitors (tofacitinib) in FMF patients [17]. Although the authors used tofacitinib to treat colchicine-resistant patients—which is not the case for our cohort—the drug suppresses disease activity and febrile attacks in patients with associated inflammatory comorbidities and chronic manifestations of the disease. Since pyrin is well known to assemble inflammasome complexes in response to perturbations [18], the overexpressed level of ASC confirms the pathogenic role of inflammasomes in FMF. However, it seems an ASC-dependent NLRP1-independent inflammasome, since the NLRP1 transcription level has not increased. Consistent with this idea, Kastner et al. demonstrated that FMF depended on caspase1, ASC, and IL1 signalling but was independent of NLRP3, NLRC4, and AIM2 [19].

In TRAPS patients, we observed a general upregulation of all pathways that certainly needs further insights. Activation of NF-κB in TRAPS is a controversial issue, as some authors demonstrated a reduction in the NF-κB signalling pathway upon expression of TRAPS-associated mutations, while many others resulted in pathway upregulation [20,21,22,23]. Further data supporting NF-ΚB activation are found in the activation of the PI3K–AKT pathway since AKT leads to IΚB-α phosphorylation and subsequent NF-κB activation [24]. GSK-3β is also important in improving the NF-κB transcriptional activity and in preventing apoptosis. MAPK (ERK and SAPK–JNK) pathway activation observed in our cohort upheld the concept of hyperinflammatory cells in TRAPS patients. Indeed, spontaneous activation of JNK and p38 MAPK has been previously reported [25]. The JAK–STAT pathway activation, instead, has not been reported yet in the literature. It will be interesting to deepen the study of this aspect in a larger cohort, and if activation is confirmed, the use of JAK inhibitors in TRAPS patients not responding to other therapies could be hypothesised. Notably, TRAPS is likely to be closely associated with the activity of the NLRP3 inflammasome, since TNF-α has been implicated in activating caspase-1 [26], as observed in our cohort. Inflammasome activation might also be sustained by proteostasis deregulation, frequently observed in TRAPS patients [27]. An examination of the heatmap (Figure 3) reveals that it has been suggested at least for the TRAPS group in which the pattern of signalling compounds may relate to individual patients. For almost all pathways, indeed, the protein expression levels of patients P9, P10, P11, and P13 can be comparable with healthy controls. Patient 9, a carrier of a mild mutation, probably owes his molecular pattern to a good response to the anti-TNF therapy (infliximab). Although he had reported occasional febrile outbreaks, infliximab apparently inhibited the disease well at the molecular level. Patients 10 and 11 instead presented intronic variants of the *TNFRSF1A* gene, reported as benign [28] and which are probably less characteristic of the disease. As for patient 13, both the particular mutation reported (deletion Y103-R104) and the anti-TNF therapy (etanercept) could be responsible for the observed molecular pattern, characterised by the solely activation of the JAK-STAT pathway. It is also interesting to note that there is a TRAPS patient (P18) who exhibited the highest levels of expression in all pathways. P18 carried the rare mutation D12E found in a few other cases in the literature that were reported to be linked to complex symptoms [29]. The observed high level of MyD88 may suggest activation of a TLR MyD88-dependent pathway that, in turn, can initiate reactive oxygen species (ROS) production, elevated in TRAPS patients [30]. This hypothesis can be supported by the increased IL1β level observed in our cohort. An exaggerated TNF-independent mitochondrial ROS production, indeed, may activate the IL-1 pathway [31].

Regarding BS, the E383K gain-of-function mutation carried by both patients led to the activation of the already-known NF-κB pathway, together with the intermediates overexpression of PI3K–AKT and MAPK (p38) pathways. Little is known about the role of these pathways in BS, mainly controversial data that do not consider any *NOD2* mutations [32]. Indeed, Zhao et al. stated that the PI3K–AKT pathway negatively regulates *wild-type* NOD2-mediated NF-κB pathway.

Nothing certain can be said of the other mAIDs poorly represented in our cohort. The few studies in the literature suggested an interesting molecular connection between the inflammasome, maybe that of the pyrin, and MKD [33,34] or that the JAK–STAT pathway could play a role in the MKD pathogenesis [35]. *NRLP12*-mutated gene (not G448A) was reported to induce a clear reduction in its inhibitory properties on NF-κB signalling [36]. We need to increase the sample size, in order to confirm the activation of all pathways for MKD and the similarity to controls observed in our NLRP12D G448A patient.

The elevated cytokine signature is a milestone of the mAIDs. Some considerations can be made on the cytokine profile of our entire cohort, in particular the upregulation of IL22 and IL23. This would clearly suggest an important role for Th17 or Th17-like cells in mAIDs, since Th17 cells, which are directly involved and mediate chronic inflammation, are characterised by the production of IL17 and IL22, as well as the recruitment of neutrophils and other inflammatory cells [37]. IL23, which promotes Th17 cell development, as well as IL17 and IL22, plays essential roles in various inflammatory diseases [38,39]. Targeting Th17 cells and their related cytokines (IL17/22/23) may be an effective therapeutic approach for autoinflammatory diseases. A linkage between Th17 cells and mAIDs has been found in cryopyrin-associated periodic syndromes (CAPS), FMF, Behçet disease, and adult-onset Still’s disease. In gene-targeted mice expressing CAPS-related mutations, the inflammasome hyperactivation resulting from excess IL1β production potentiates Th17-dominant immune responses [40]. Ovadia et al. suggest the presence of a heightened Th17 response in FMF, related to frequent attacks and FMF genotype [41]. The upregulation of Th17 shows a correlation with the progression of Behçet [42] and Still’s diseases [43]. A better understanding of the involvement and possible pathogenic role of Th17 cells in mAIDs requires; however, further investigation and a larger cohort of patients. Moreover, if the different cytokine pattern for the three mAIDs considered (FMF, TRAPS, and BS) is confirmed in future studies, this could become a further way of stratifying those autoinflammatory conditions that are difficult to classify.

There were some limitations in our study—namely, the small sample size in these clinically heterogeneous diseases and little clinical information about the patients. Hence, further research should include more comprehensive and larger cohorts of mAIDs. Analysis of the significant molecules from signalling pathways with biostatistical models will also be required to generate specific diagnostic algorithms and to identify valuable biomarkers to be used as targets for specific therapeutic interventions.

## 4. Materials and Methods

### 4.1. Subjects

A total of 37 subjects, originating from the Veneto Region, a northeast Italian region, were enrolled. The study was approved by the Local Ethics Committee of the University-Hospital of Padova, and all subjects gave their full informed written consent at enrolment.

Cases were 7 FMF patients (3 males, 4 females; mean age ± SD: 37.4 ± 12.3 years), 12 TRAPS patients (6 males, 6 females; mean age ± SD: 39.2 ± 15.1 years), 1 MKD patient (male, 14 years old), 2 BS patients (females; mean age ± SD: 46.5 ± 17.7 years) and 1 NLRP12D patient (male; 38 years old). Healthy controls were 14 donors (5 males, 9 females; mean age ± SD: 38.3 ± 8.9 years).

Detailed clinical characteristics of the patients at the time of the enrolment are shown in Table 1. The majority of them presented mild genetic mutations associated with their diseases; thus, the diagnosis was also strongly based on clinical criteria. Additionally, only 6/37 patients were not under treatment. The disease symptoms were reported under control by most of the patients at the time of blood withdrawal.

### 4.2. Serum and Peripheral Blood Mononuclear Cells Samples

PBMCs and serum were collected from both patients and controls. PBMCs were seeded at a density of 106 cell/mL and cultured for 24 h, to overcome the possible interference of therapy. The cells were washed with ice-cold PBS and lysed in 200 µL of RIPA buffer (Pierce, Thermo Fisher Scientific, Waltham, MA, USA) containing protease and phosphatase inhibitors and 5 U/mL Benzonase (Sigma Aldrich, St. Louis, MO, USA). Protein concentrations of these lysates were determined by BCA protein assay (Pierce, Thermo Fisher Scientific).

### 4.3. Reverse Phase Protein Array (RPPA)

Lysates were solubilised in 4 × SDS loading buffer and heated for 5 min at 95 °C. RPPA assays of the components of the NF-κB, PI3K–Akt, MAPK, JAK–STAT–c-Sr, and inflammasome (NALP1) signalling pathways were performed as previously described [10]. 

Briefly, samples were spotted in duplicates onto a nitrocellulose-coated glass slide (Grace Bio-labs, Bend, OR, USA) using a microarray robot (MicroGrid 610, Digilab, Marlborough, MA, USA). The printed slides were blocked and incubated with specific primary antibodies listed in Table 2 (Cell Signalling) overnight at 4 °C, with shaking. Β actin was included as a housekeeping protein, to control protein loading. After incubation with infrared secondary antibodies (800 CW LI-COR anti-rabbit antibody and 700 CW LI-COR anti-mouse antibody), the slides were scanned with a Licor Odyssey scanner (LI-COR, Biosciences) at 21 um resolution at 700 nm and 800 nm. The fluorescent data were processed with GenePix Pro-6 Microarray Image Analysis software (Molecular Services Inc., San Diego, CA, USA). Protein signals were determined with background subtraction and normalisation to the internal housekeeping targets using the RPP analyser [44].

### 4.4. Antibody Microarray

Profiling of IL-1β, IL-6, IL-8, TNF-α, IL-17, IL-22, IL-23 was performed on serum samples using an array-based assay previously described [45]. Specifically, captured antibodies (R&D) were diluted in printing buffer (50 mM Trehalose, 1X PBS, 0.01% Tween-20) and spotted onto poly-l-lysine-coated glass slides (Sigma Aldrich) by using a microarray robot (MicroGrid II, BioRobotics Inc., Redwood City, CA, USA). The slides were blocked, washed, and incubated for 1 h, with the sera and a cocktail of standard antibodies serial diluted in a reagent diluent (R&D) with an initial concentration of 1000 pg/mL. After the washing step, a mix of detection antibodies (R&D), diluted in the reagent diluent following the manufacturer’s instructions, was added to each slide and incubated for 1 h. An additional tyramide amplification step added Streptavidin-HRP (BioRad, Hercules, CA, USA) at 1:1000 for 15 min and BioRad amplification unit mix before washing step and incubation with Streptavidin Cy5 (Life Technology, Carlsbad, CA, USA) at 1:1000 in 3% BSA. The slides were dried by centrifugation at 1200× *g* for 5 min and scanned with Axon 4200 AL scanner at 635 nm. Cy5 fluorescence, gained from the microarray system, would be quantified using GenePix Pro-6 software (Axon Instrument, Scottsdale, AZ, USA), subtracted from the background, and used for computing average and standard deviation. The concentrations of each cytokine were calculated based on a standard curve of known amounts of each cytokine.

### 4.5. Statistical Analysis

Wilcoxon matched-pairs test using GraphPad Prism was applied to compare the level of expression of signalling molecules in patients and controls, obtained by two independent experiments. Between-group comparisons of cytokines expression levels were performed with non-parametric tests (Mann–Whitney). A two-sided p value less than 0.05 was considered statistically significant.

## 5. Conclusions

More generally, the present study draws attention to the importance of inflammatory signalling pathways and cytokine production in the development of mAIDs. The generalised and constitutive increase in protein levels for several and some novel pro-inflammatory signalling factors and cytokines give rise to the valuable protein microarray assay for mAIDs screening for pathogenic and therapeutic biomarkers. In this sense, it would be interesting to analyse the mRNA level of these mediators to fully analyse their expression and production. The different patterns of pathways’ activation reveal how variable clinical phenotypes are influenced not only by the genetic background but also by complex molecular mechanisms. Our results indicate a previously unrecognised role for inflammatory pathways in some mAIDs, such as JAK–STAT signalling in TRAPS, and the production of Th17-related cytokines that pave the way for the study of other noncanonical autoinflammatory pathways and the role of Th17 cells in autoinflammatory diseases.

## Figures and Tables

**Table 1:** Characteristics of the 23 mAIDs patients at the time of enrolment.

Patient	Age	Sex	Disease	Associated Gene	Mutations or Polymorphisms	Clinical	Treatments
P1	30 y	F	FMF	*MEFV*	E148Q he	Absence of febrile episodes and arthralgia; CRP and SAA slightly above the normal limits	None; (Colchicine 1mg/day discontinued for remission)
P2	48 y	F	FMF	*MEFV*	E148Q he + M680I he	Absence of febrile episodes, arthralgia, and abdominal pain; CRP and SAA normal	Colchicine 1 mg/day
P3	38 y	M	FMF	*MEFV*	R202 Q he	Occasional febrile episodes not associated with other symptoms; CRP and SAA slightly above the normal limits	Colchicine 1 mg/day
P4	19 y	F	FMF	*MEFV*	none	Occasional febrile episodes not associated with other symptoms	Colchicine 1 mg/day
P5	34 y	F	FMF	*MEFV*	E148Q he + P369S he	Accentuation of joint symptoms in the ankles and stabbing pain in the thoracic area; CRP and SAA normal; a slight reduction in neutrophils	Colchicine 1 mg/day
P6	57 y	M	FMF	*MEFV*	R202Q he	Abdominal pain; occasional febrile episodes	Colchicine 1mg/day
P7	36 y	M	FMF	*MEFV*	M641I he	Absence of febrile episodes and arthralgia	none
P8	70 y	M	TRAPS	*TNFRSF1A*	S59P he	Episodes of arthralgia	Steroids 4mg
P9	46 y	F	TRAPS	*TNFRSF1A*	R92Q he	Pain in the cervical spine; occasional febrile episodes; arthralgia	Infliximab 5 mg/kg every 6 weeks
P10	22 y	M	TRAPS	*TNFRSF1A*	c.626-32G > T	Abdominal pain; occasional febrile episodes, arthralgia; CRP slightly above the normal limit	Steroids as needed
P11	27 y	F	TRAPS	*TNFRSF1A*	c.625+10A > G	Hand dermatitis; no episodes of hyperpyrexia; no skin rash; no oral or genital ulcers; not arthralgia.	Colchicine 1mg/day; NSAIDs
P12	36 y	F	TRAPS	*TNFRSF1A*	V95M he	Occasional febrile episodes not associated with other symptoms	Canakinumab 150mg/8 weeks
P13	34 y	M	TRAPS	*TNFRSF1A*	delY103-R104	Controlled articular and cutaneous manifestations	Etanercept 50mg/week
P14	32 y	M	TRAPS	*TNFRSF1A*	T50M he	Controlled disease manifestations	none
P15	54 y	F	TRAPS	*TNFRSF1A*	R104Q he	Occasional febrile episodes	Anakinra 100mg/day
P16	41 y	M	TRAPS	*TNFRSF1A*	T50M he	Controlled articular and cutaneous manifestations	Anakinra 100mg/day
P17	34 y	M	TRAPS	*TNFRSF1A*	R92Q he	Absence of febrile episodes and arthralgia	none
P18	18 y	F	TRAPS	*TNFRSF1A*	D12E he	Controlled disease manifestations	Anakinra 100mg/day
P19	56 y	F	TRAPS	*TNFRSF1A*	R92Q he	Controlled disease manifestations	Anakinra 100mg/day
P20	14 y	M	MKD	*MVK*	V377I ho	Controlled articular and cutaneous manifestations	none
P21	59 y	F	BS	*NOD2*	E383K he	Controlled articular and cutaneous manifestations; severe uveitis	Steroids 4mg; Humira 40mg/14days
P22	34 y	F	BS	*NOD2*	E383K he	Controlled articular and cutaneous manifestations; progressive uveitis	none; (Steroids 4mg discontinued for remission)
P23	38 y	M	NLRP12D	*NLRP12*	G448A he	Absence of febrile episodes and arthralgia	none

y = years old; M = male; F = female; TRAPS = TNF-receptor associated periodic syndrome, FMF = familial mediterranean fever; BS = Blau syndrome; MKD = mevalonate kinase deficiency; he = heterozygous; ho = homozygous; SAA = serum amyloid A; CRP = C reactive protein.

**Table 2:** List of primary antibodies used in this inflammatory signalling study with RPPA.

NF-κb Pathway		PI3/AKT Pathway		MAPK Pathways	
Antibody	Dilution	Antibody	Dilution	Antibody	Dilution
NF-κB	1:250	p eNOS (Ser1177)	1:500	p MKK3/MKK6 (Ser189/Ser207)	1:100
p NF-κB p65 (Ser536)	1:100	p BAD (Ser136)	1:500	p P38 MAPK (Thr180/Tyr182)	1:1000
IΚBα	1:100	p AKT (Thr308)	1:50	p SAPK/JNK (Thr183/Tyr185)	1:1000
p IΚBα (Ser32)	1:500	p AKT (Ser473)	1:25	TRAF2	1:500
IKKα	1:250	PI3K p100α	1:250	TRAF6	1:500
IKKβ	1:250	PI3K p85	1:250	p HSP27 (Ser82)	1:50
p IKKα/β (Ser176/Ser177)	1:250	p PDK1 (Ser259)	1:1000	p c-JUN (Ser63)	1:200
IKKγ	1:250	p GSK 3β (Ser9)	1:500	p ERK1/2 (Thr202/Tyr204)	1:2000
p IRAK1 (Thr209)	1:3000	p c-RAF (Ser259)	1:1000	MEK1/2	1:250
A20	1:150	p PTEN (Ser380)	1:1000	p MEK1/2 (Ser217/Ser221)	1:250
				p SEK1/MKK4 (Ser257/Thr261)	1:250
				MKK7	1:100
				p TAK1 (Ser412)	1:100
				p ATF2 (Thr71)	1:250
**SRC–JAK–STAT3 pathway**		**INFLAMMASOME pathway**		**others**	
**Antibody**	**Dilution**	**Antibody**	**Dilution**	**Antibody**	**Dilution**
p STAT3 (Tyr705)	1:100	IL-1β (*)	1:100	MyD88	1:50
p c-SRC (Tyr416)	1:1,0000	ASC (TMS1)	1:150	Actin (Mouse)	1:1000
SOCS3	1:500	CASPASE1	1:250	Actin (Rabbit)	1:1000
SHIP2	1:2000	CLEAVED CASPASE1	1:50		
p RIP2 (Ser176)	1:500	NALP1	1:150		
p JAK2 (Tyr1007)	1:1000	p BAD (Ser136)	1:500		

All of the listed primary antibodies were produced in rabbit except (*) IL-1β produced in mouse. The primary antibodies were purchased from Cell Signalling Technology ^®^ (Topsfield, MA, USA).

## Data Availability

The data presented in this study are available on request from the corresponding author. The data are not publicly available due to privacy restrictions.

## Supplementary Materials

The following supporting information can be downloaded at: https://www.mdpi.com/article/10.3390/ijms23031828/s1.
