# Supplementary material for: A Pro-Inflammatory Signature Constitutively Activated in Monogenic Autoinflammatory Diseases
Source: Int J Mol Sci. 2022 Feb 5;23(3):1828. doi: 10.3390/ijms23031828 (PMC8836675; doi:10.3390/ijms23031828)
Supplement: Supplementary file 1 [file ijms-23-01828-s001.zip › ijms-1546167-supplementary.pdf]

International Journal of *Molecular Sciences*

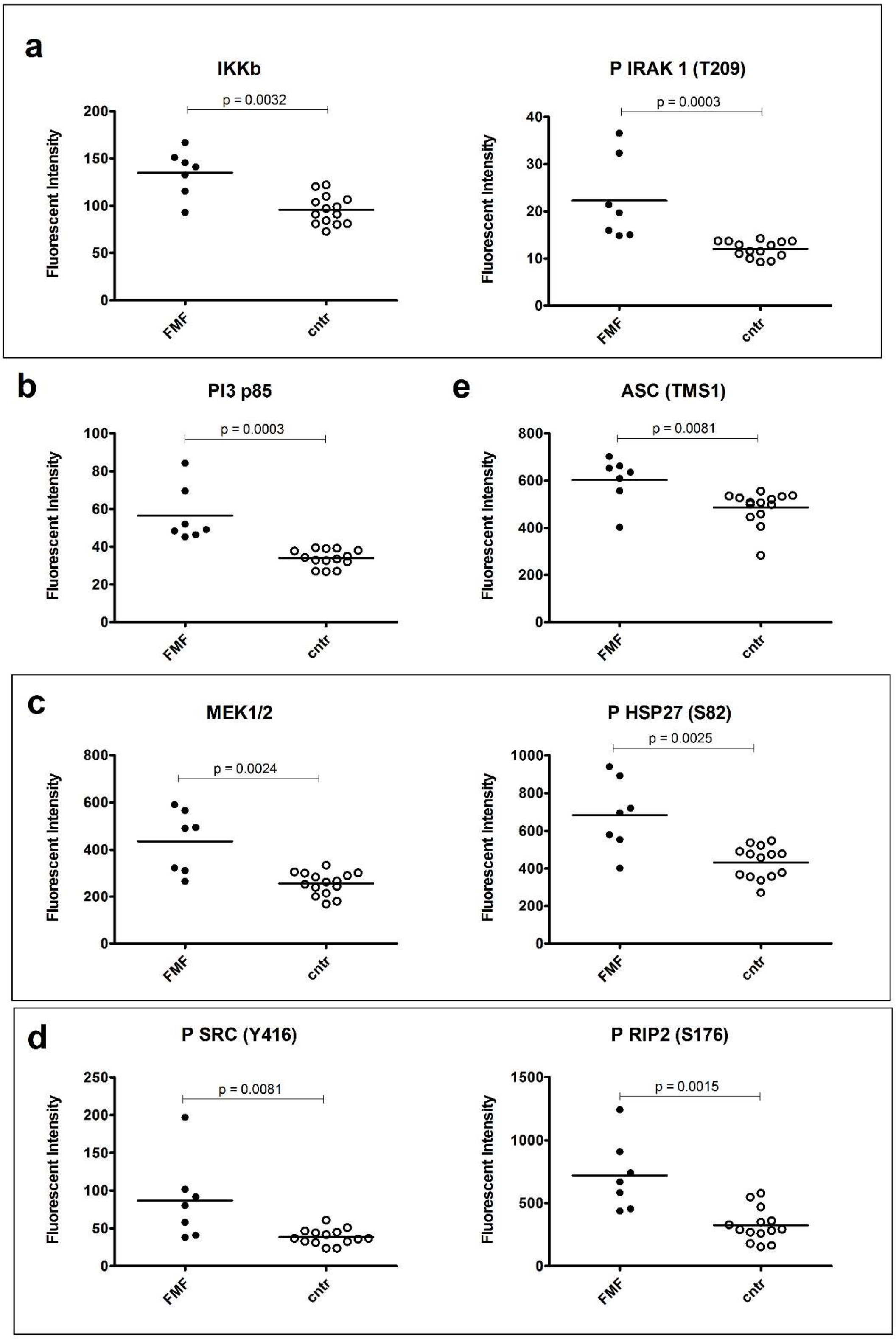

Figure S1. Scatter plots of the significantly upregulated expression levels of all intermediates in FMF patients from (a) NF-κB pathway, (b) PI3K–Akt pathway, (c) MAPK pathway, (d) JAK–STAT pathway, and (e) NLRP1 inflammasome pathway. All fluorescent signals are reported as arbitrary fluorescence units (AFU), with β-actin signal normalisation. Data are shown as means ± SD of three independent experiments.

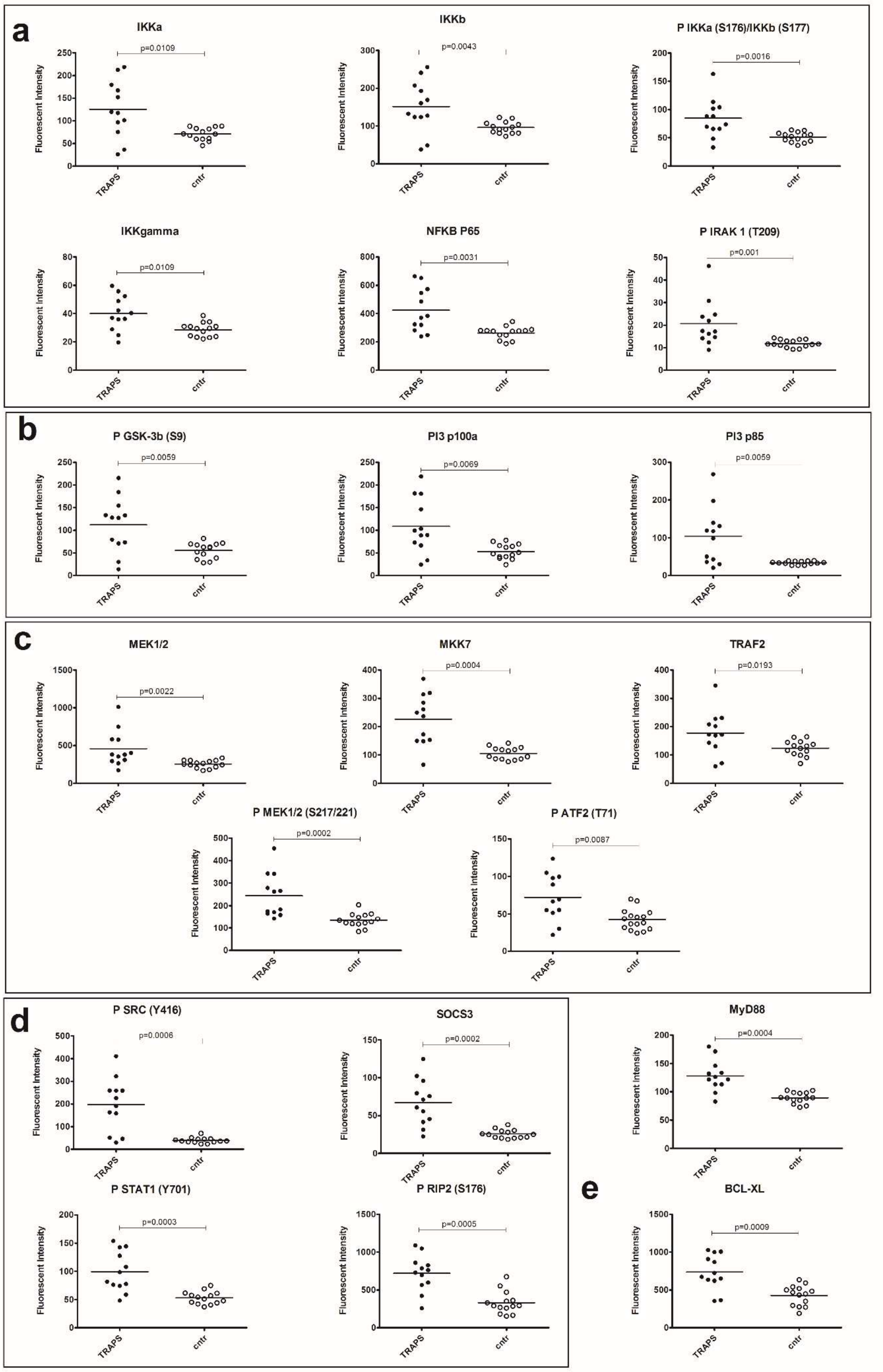

Figure S2. Scatter plots of the significantly upregulated expression levels of all intermediates in TRAPS patients from (a) NF-κB pathway, (b) PI3K–Akt pathway, (c) MAPK pathway, (d) JAK–STAT pathway, and (e) NLRP1 inflammasome pathway. All fluorescent signals are reported as arbitrary fluorescence units (AFU), with β-actin signal normalisation. Data are shown as means ± SD of three independent experiments.

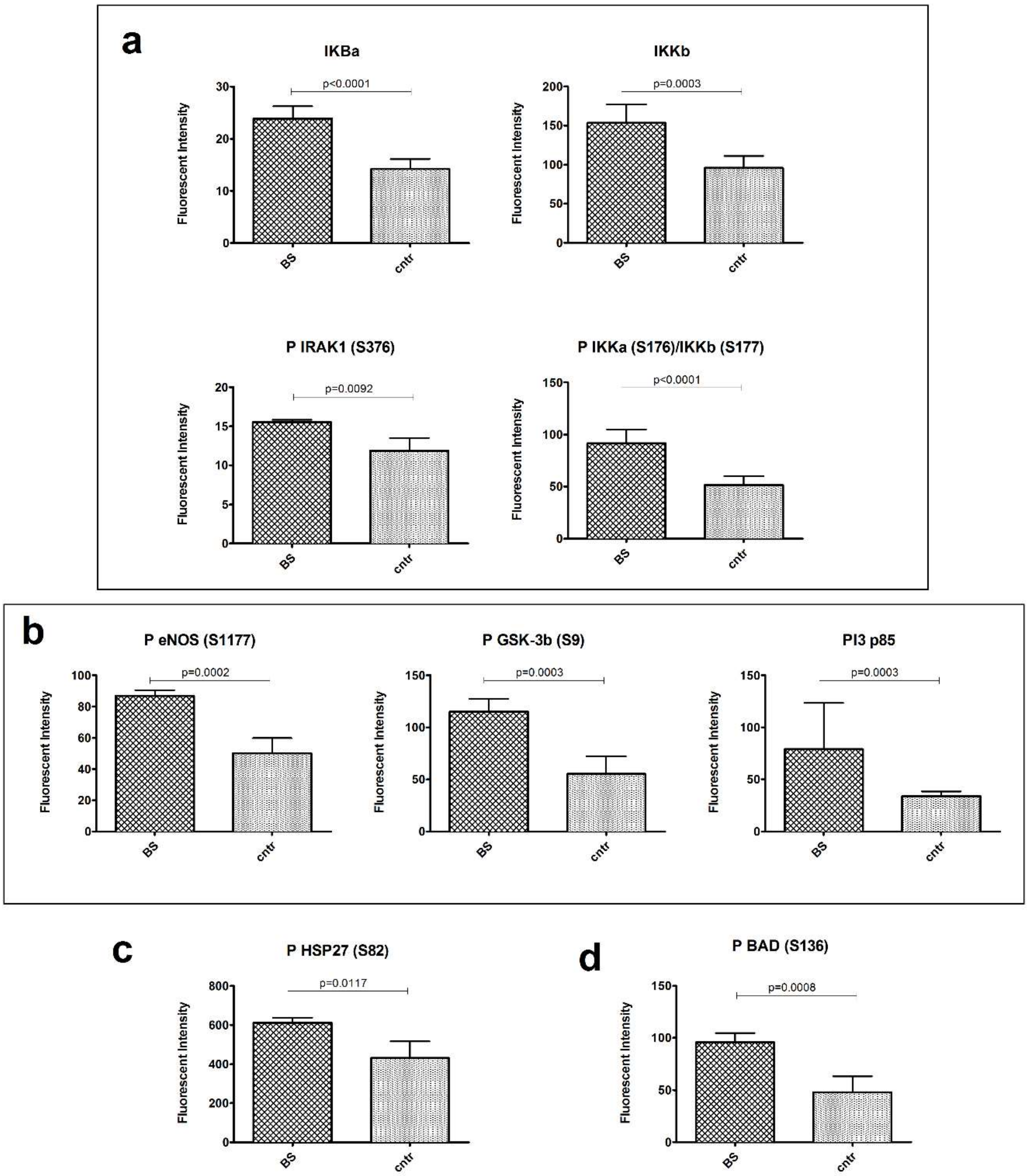

Figure S3. Scatter plots of the significantly upregulated expression levels of all intermediates in BS patients from (a) NF-κB pathway, (b) PI3K–Akt pathway, (c) MAPK pathway, and (d) NLRP1 inflammasome pathway. All fluorescent signals are reported as arbitrary fluorescence units (AFU), with β-actin signal normalisation. Data are shown as means ± SD of three independent experiments.
